# The Sufferings of Christ

**Published:** 1888-02-18

**Authors:** 


### Words of Consolation.

[Communications for this column should be addressed "Chaplain," care of the Editor of The Hospital, who will gratefully welcome any suggestions or inquiries from matrons, nurses, and the staff generally.]

### LXVI.—The Sufferings of Christ.

*For Reading to the Sick.*

From the consideration of our Lord's bodily sufferings we pass now to the darkest passage of His tremendous sacrifice—the last three hours upon the cross. "Now from the sixth hour there was darkness over all the land until the ninth hour. And about the ninth hour Jesus cried with a loud voice, saying, Eli, Eli, lama sabachthani? that is to say, My God, my God, why hast thou forsaken me?" (Matthew xxvii. 45, 46.) The outer darkness, nature mourning over the crucifixion of her King and her God, was but the type of surroundings yet more awful in the spiritual kingdom. Through these our Redeemer passed, working out in the depth of the unknown sufferings the completion of the great atonement. In venturing so far as we dare into this darkness, the central thought to be kept in mind is "desertion." Sin is desertion of God. Hell is desertion by God. Therefore in the depths of the atonement we should look for something corresponding. We find the sense of desertion expressed in the cry, "My God, my God, why hast thou forsaken me?"

Here was the taste of death, of real death. In the feeling of being forsaken He tasted of the very essence and reality of death; far more so than when He calmly breathed forth His spirit into the Father's hands, once more in consciousness of the light and peace of Heaven. The latter experience was *dissolution*, but the former was *desolation*. Here lies a most important and instructive distinction. There are two kinds of death spoken of in Holy Scripture, the first and the second death. Both are the consequences of sin. From the first of these we are not yet delivered. The sacrifice of the cross has not made any difference yet to the fact of the first death. It has taken away much of the terror which surrounds it. We are shown how to die, and how to bear all that is painful in connexion with dissolution; but it has not in any sense saved us from paying this penalty as the first consequence of sin. The first death is nevertheless a clue to the meaning of the second. Dissolution is the separation of body from soul. The desolation of Hell, the outer darkness, consists in the separation of body and soul from God. Adam and Eve paid this penalty in a figure before their natural death: "In the day that thou eatest thereof thou shalt surely die" (Genesis ii. 17). What was the penalty immediately incurred? Separation: separation from Paradise, from the tree of life. They were "cut off" in the desolating abiding sense of God's displeasure at sin long before their physical powers were decayed or their natural force abated. Now, as the phrase "cut off" is used so often in the Bible to express the necessary result of sin, it becomes plain that the separation of the soul that sinneth is the very essence of the bitter fruit of disobedience. "That soul shall be cut off from my presence" (Leviticus xxii. 31) is the constant burden of Old Testament warnings. You will readily associate this very common phrase with the words applied by the prophet to our Lord's sacrifice: "He was cut off out of the land of the living" (Isaiah liii. 8). It was in the cutting off which He was permitted to feel that He identified Himself so very closely with the curse due unto sin. So that in the great darkness of Calvary we can discern somewhat the same lesson being taught us about sin as a whole as we have observed in detail. The sacrifice of that love, while supplying the best motive for penitence, gives the plainest forecast of the dreadful retribution in store for all who prefer darkness to light.

### DEAD BY THE WAY.

I saw him sink on the dusty road
  With the dead and dying, a ghastly throng;
I may have lightened the weary load
  He carried a little—my arms were strong—
My bosom echoed his last faint sigh;
  But war is war, and the trumpets bray,
And we march though the best and the bravest lie
  Unnamed, unnoticed, just dead by the way.

Had I raised a stone that might bear his name,
  What good? I was nameless as well as he.
'Tis the deeds that are done swell the note of fame,
  Not those we dream of as yet to be.
I could but cover his sweet, still face,
  And marvel his heart so calmly lay,
And pray, as I stepped to the ranks in his place,
  I might fall in the battle, and not by the way.

—*Hugh Conway*

